# Molecular characterization and *In Vitro* synthesis of infectious RNA of a Turnip vein-clearing virus isolated from *Alliaria petiolata* in Hungary

**DOI:** 10.1371/journal.pone.0224398

**Published:** 2019-10-24

**Authors:** Tamás Tóth, Péter Gyula, Pál Salamon, Szilvia Kis, Anita Sós-Hegedűs, György Szittya

**Affiliations:** 1 Department of Plant Biotechnology, Agricultural Biotechnology Institute, National Agricultural Research and Innovation Center, Gödöllő, Hungary; 2 Department of Genetics, Agricultural Biotechnology Institute, National Agricultural Research and Innovation Center, Gödöllő, Hungary; Oklahoma State University, UNITED STATES

## Abstract

A tobamovirus was isolated from leaves of *Alliaria petiolata* plants, showing vein-clearing, interveinal chlorosis, and moderate deformation. Host range experiments revealed a high similarity of isolate ApH both to ribgrass mosaic viruses and turnip vein-clearing viruses. The complete nucleotide sequence of the viral genome was determined. The genomic RNA is composed of 6312 nucleotides and contains four open reading frames (ORF). ORF1 is 3324 nt-long and encodes a polypeptide of about 125.3 kDa. The ORF1 encoded putative replication protein contains an Alphavirus-like methyltransferase domain. ORF2 is 4806 nt-long and encodes a polypeptide of about 182 kDa. The ORF2 encoded putative replication protein contains an RNA-dependent RNA polymerase, catalytic domain. ORF3 encodes the putative cell-to-cell movement protein with a molecular weight of 30.1 kDa. ORF4 overlaps with ORF3 and encodes the coat protein with a size of 17.5 kDa. Sequence comparisons revealed that the ApH isolate has the highest similarity to turnip vein-clearing viruses and should be considered an isolate of Turnip vein-clearing virus (TVCV). This is the first report on the occurrence of TVCV in Hungary. *In vitro* transcripts prepared from the full-length cDNA clone of TVCV-ApH were highly infectious and induced typical symptoms characteristic to the original isolate of the virus. Since infectious clones of TVCV-ApH and crTMV (another isolate of TVCV) markedly differed in respect to recovery phenotype in *Arabidopsis thaliana*, it is feasible to carry out gene exchange or mutational studies to determine viral factors responsible for the symptom recovery phenotype.

## Introduction

The genus Tobamovirus (family Virgaviridae) represents a group of rod-shaped (approx. 300–310 nm long) plant viruses with single-stranded, undivided positive-sense RNA genomes of approximately 6400 nucleotides. There are currently 37 species in this genus including the type species Tobacco mosaic virus (TMV) which is one of the best-studied and characterized plant viruses [1]. The genome of tobamoviruses contains at least four ORFs. ORF1 codes for the 124–132 kDa small replicase subunit, while the suppression of an amber termination codon of ORF1 results in the 181–189 kDa large replicase subunit (ORF2). The ORF3 and ORF4 translate from 3’ subgenomic RNAs and result in a 28–31 kDa movement protein, and a 17–18 kDa coat protein, respectively [2]. Several reports demonstrated that besides its role in replication, the p124–132 protein acts as a viral silencing suppressor through the binding of viral siRNAs [3–5].

The genus Tobamovirus is divided at least into three subgroups based on natural host range, genomic organization and phylogenetic clustering of isolates [6–8]. The Subgroup 3 includes crucifer- and plantain-infecting viruses and currently comprises the following four species: Ribgrass mosaic virus (RMV), Turnip vein-clearing virus (TVCV), Youcai mosaic virus (YoMV) formerly designated as Oilseed rape mosaic virus (ORMV) and Wasabi mosaic virus (WMoV) [1]. TVCV was first identified from infected turnip (*Brassica rapa* L.) plants in Oklahoma [9] as a contaminant of a preparation of cauliflower mosaic virus (CaMV). At the time that TVCV was first being characterized by Lartey *et al*., (1993) [9,10] a related virus isolate was also being described by Dorokhov *et al*., [11–13]. Dorokhov *et al*. designated their isolate as crTMV (for crucifer TMV), however, it differs only 6.5% in the nucleotide sequence from the TVCV isolate [14] and therefore it is regarded as an isolate of TVCV [15].

In this work, we determined the complete nucleotide sequence of the genomic RNA of TVCV isolated from *Alliaria petiolata* in Hungary and report the preparation of the infectious cDNA clone of the virus.

## Materials and methods

### Collection, host range determination, and maintenance of the virus

The virus was isolated from *Alliaria petiolata* in Hungary and maintained in *Nicotiana benthamiana* until an infectious transcript was created. The host range was studied by inoculating the following test plants: *Arabidopsis thaliana* Col-0 and Bur-0 ecotypes, *Brassica chinensis*, *Brassica rapa* var. rapa, *Capsicum annuum*, *Chenopodium murale*, *Chenopodium quinoa*, *Datura stramonium*, *Nicotiana benthamiana*, *Nicotiana glutinosa*, *Nicotiana megalosiphon*, *Nicotiana sylvestris*, *Nicotiana tabacum* cv. Samsun, *Nicotiana tabacum* cv. Xanthi, *Ocimum basilicum*, *Solanum betaceum*, *Solanum lycopersicum*.

### Cloning of the virus and the making of an infectious transcript

Virions and viral genomic RNA were purified from infected *Nicotiana benthamiana* plants according to a tobamovirus isolation protocol [16]. The viral RNA was converted to cDNA by SuperScript IV reverse transcriptase (Invitrogen) using the virus-specific primer RMV 3 (5’-TGG GCC CCT ACC CGG GGT TAG GGA GG-3’). The viral genome was amplified with CloneAmp^™^ HiFi PCR Premix (Clontech) in one piece using the primers RMV Inf Fw (5’-CGG TAC CCG GGG ATC GTT TAG TTT TAT TGC AAC AAC AAC A-3’) and RMV Inf Rev (5’-CGA CTC TAG AGG ATC TGG GCC CCT ACC CGG GGT-3’). The PCR products were cloned into pUC19 vector using the In-Fusion® HD Cloning Kit (Clontech). The cloned viral genome was sequenced by the Sanger method in eight parts and the sequences were assembled by CAP3 [17]. The homology search was performed using the BLAST server [18]. The full-length viral sequence was deposited in GenBank under the accession number MH370485.

The infectious transcript was created in the following way: a PCR was carried out with a primer specific to the 5’ end of the virus-containing a T7 promoter as a 5’ flag (RMV 5’ T7 BamHI, 5’-GAG AGG ATC CTA ATA CGA CTC ACT ATA GGT TTA GTT TTA TTG CAA CAA CAA-3’), and another primer specific to a region in the virus containing a natural *Stu*I recognition site (RMV 632 minus, 5’-ATC AGT ACC GTT CGC CGT CG T-3’). The PCR product was phosphorylated with T4 polynucleotide kinase (Thermo Scientific), digested with *Stu*I, and ligated into the pUC19-TVCV vector that was first *Kpn*I digested, blunted with T4 DNA polymerase, digested with *Stu*I, and then dephosphorylated with Antarctic phosphatase (New England Biolabs). The 3’ end of the virus was edited to include a *Bst*XI restriction enzyme recognition site to allow linearization of the vector for *in vitro* transcription. For this, the 3’ end of the virus was amplified with TVCV MP fw (5’-CGG GTT GGC AGC CGT TAG CTC TGG-3’) and TVCV 3’ BstXI (5’-TAG CCA ATT ATT TGG GCC CCT ACC CGG GGT TAG GGA GG-3’) primers, the product was phosphorylated with T4 polynucleotide kinase, cut with *Bst*EII, and ligated into the pUC19-T7::TVCV that was cut with *Sph*I, blunted with T4 DNA polymerase, cut with *Bst*EII, and dephosphorylated with Antarctic phosphatase. The positive clones were verified by Sanger sequencing. For the *in vitro* transcription, the pUC19-T7::TVCV-BstXI vector was linearized with *Bst*XI, and transcribed with T7 RNA polymerase either with or without the 7mG cap analog (New England Biolabs) as described earlier [19].

### Inoculation of plants

For every leaf, 4 μl of the *in vitro* transcription reaction mixture was combined with 3.5 μl water and 7.5 μl 2× inoculation buffer containing 50 mM glycine, 30 mM K₂HPO₄ pH 9.2, 1% (w/v) Bentonite-SF (Serva), 1% (w/v) Celite® 512 (Fluka). The inoculation solution was applied onto the leaves of four-week-old *Nicotiana benthamiana* plants and rub inoculated with a glass spatula. After inoculation, the plants were sprayed with water. For the inoculation of *Arabidopsis thaliana* plants, 2 μl (1 μg) of total RNA purified from TVCV-ApH or crTMV infected systemic leaves of *N*. *benthamiana* was combined with 5.5 μl water and 7.5 μl 2× inoculation buffer and was rub inoculated into the true leaves of four-week-old *Arabidopsis* plants. Three leaves were inoculated per plant. After inoculation, the plants were sprayed with water.

### Total RNA purification and northern blot analysis

Total RNA from plants was purified using a phenol-chloroform extraction method [20]. A probe recognizing the 3’-end of the virus was amplified with primers TVCV MP fw and TVCV 3’ BstXI (see above). The labeling and the northern blot analysis were carried out according to Baksa *et al*., 2015 [21].

### Protein purification and PAGE

Proteins were purified as described earlier [22] and separated by electrophoresis on 12% TGX Stain-Free^™^ FastCast^™^ Acrylamide Gels (Bio-Rad) using ProSieve QuadColor^™^ protein marker (4.6–300 kDa) (Lonza).

### Phylogenetic analysis

The full-length genomes of tobamoviruses were retrieved from the NCBI Reference Sequence Database [23] and aligned with the MUSCLE algorithm [24]. The phylogenetic relationship was inferred using the Neighbor-Joining method [25] as implemented in the MEGA X software [26]. The accuracy of the presented tree structure was estimated by the bootstrap method (1000 replicates) [27]. The trees were drawn to scale, with branch lengths in the same units as those of the evolutionary distances used to infer the phylogenetic tree. The evolutionary distances were computed using the LogDet (Tamura-Kumar) method [28] and are in the units of the number of base substitutions per site. The rate variation among sites was modeled with a gamma distribution (shape parameter = 0.05). The differences in the composition bias among sequences were considered in evolutionary comparisons. All positions containing gaps and missing data were eliminated.

## Results and discussion

### Isolation and host range determination of the virus

**Fig 1:**
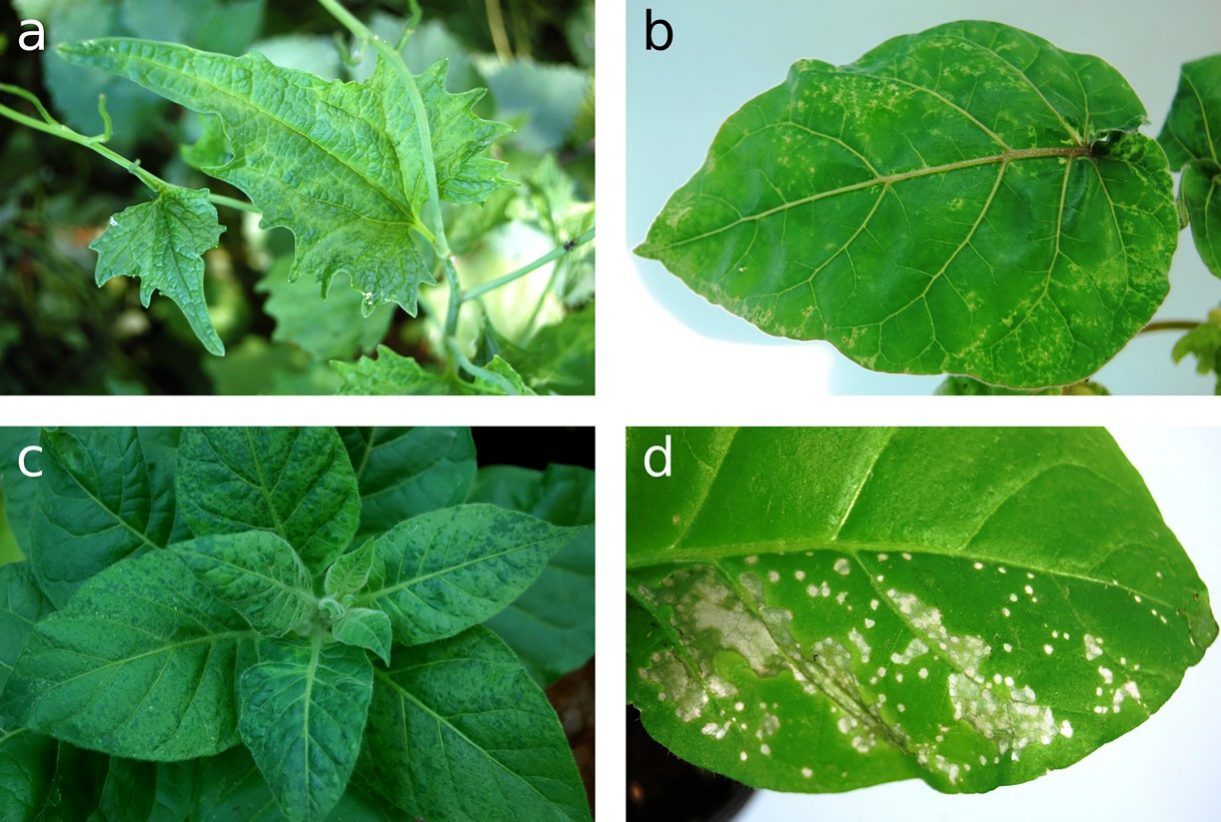
Symptoms observed in different plants infected by TVCV-ApH. (a) Mild vein clearing and mosaic in the leaf of garlic mustard (*Alliaria petiolata*) the source of TVCV-ApH. (b) Systemic symptoms on *Solanum betaceum*. (c) *Nicotiana tabacum* cv. Samsun, systemic symptoms. (d) Local lesions on *Nicotiana tabacum* cv. Xanthi-nc.

The *Alliaria petiolata* plant showing vein-clearing, chlorotic regions, and moderate leaf deformation (Fig 1) was collected at a roadside in Velence, Hungary. Several test plants inoculated by its leaf extract reacted either with local or with local and systemic symptoms characteristic to tobamoviruses. A single lesion subculture of the isolated virus caused symptoms on selected test plants (Fig 1 and Table 1) very similar to those of RMV, the only tobamovirus known to be pathogenic to cruciferous plants and *Solanum betaceum* in Hungary.

**Table 1:** Host range and symptomatology of TVCV-ApH.

Plant species	Symptoms	
	Local	Systemic
*Arabidopsis thaliana* Col-0	ns	d (+)
*Arabidopsis thaliana* Bur-0	ns	d (+)
*Brassica chinensis*	ns	mo, re (+)
*Brassica rapa* var. rapa	csp	vc, mo (+)
*Capsicum annuum*	csp	ns (-)
*Chenopodium murale*	nl	ns (-)
*Chenopodium quinoa*	cnl	ns (-)
*Datura stramonium*	nl	ns (-)
*Nicotiana benthamiana*	csp	y, d, stn, dth (+)
*Nicotiana clevelandii*	csp	chl, d, mo (+)
*Nicotiana glutinosa*	nl	ns (-)
*Nicotiana megalosiphon*	nl	ns(-)
*Nicotiana sylvestris*	nl	ns (-)
*Nicotiana tabacum* cv. Samsun	csp	mo (+)
*Nicotiana tabacum* cv. Xanthi-nc	nl	ns (-)
*Solanum betaceum*	ns	d, mo (+)
*Solanum lycopersicum* cv. Moneymaker	ns	ns (-)

csp = chlorotic spots, ns = no symptoms, nl = necrotic lesions, cnl = chlorotic-necrotic lesions, vc = vein clearing; mo = mosaic; y = yellowing, d = deformations, stn = stem necrosis, dth = death; (+) = the virus was detected by bioassays to *N*. *glutinosa*; (-) = no virus was detected by bioassays to *N*. *glutinosa*

### Cloning and sequencing of the virus

To further characterize the ApH isolate, virions were purified from systemically infected *N*. *benthamiana* leaves [16]. RNA extraction and genome amplification by RT-PCR, cloning, sequencing, the assembly of the full genome sequence and gene translations of the ApH isolate were conducted as described in the materials and methods section. A sequence homology search with the full-length genomic sequence revealed that the closest relatives of the ApH isolate are various isolates of Turnip vein-clearing virus and Ribgrass mosaic virus.

### Phylogenetic relationship of the ApH isolate and other members of the Tobamovirus family

**Fig 2:**
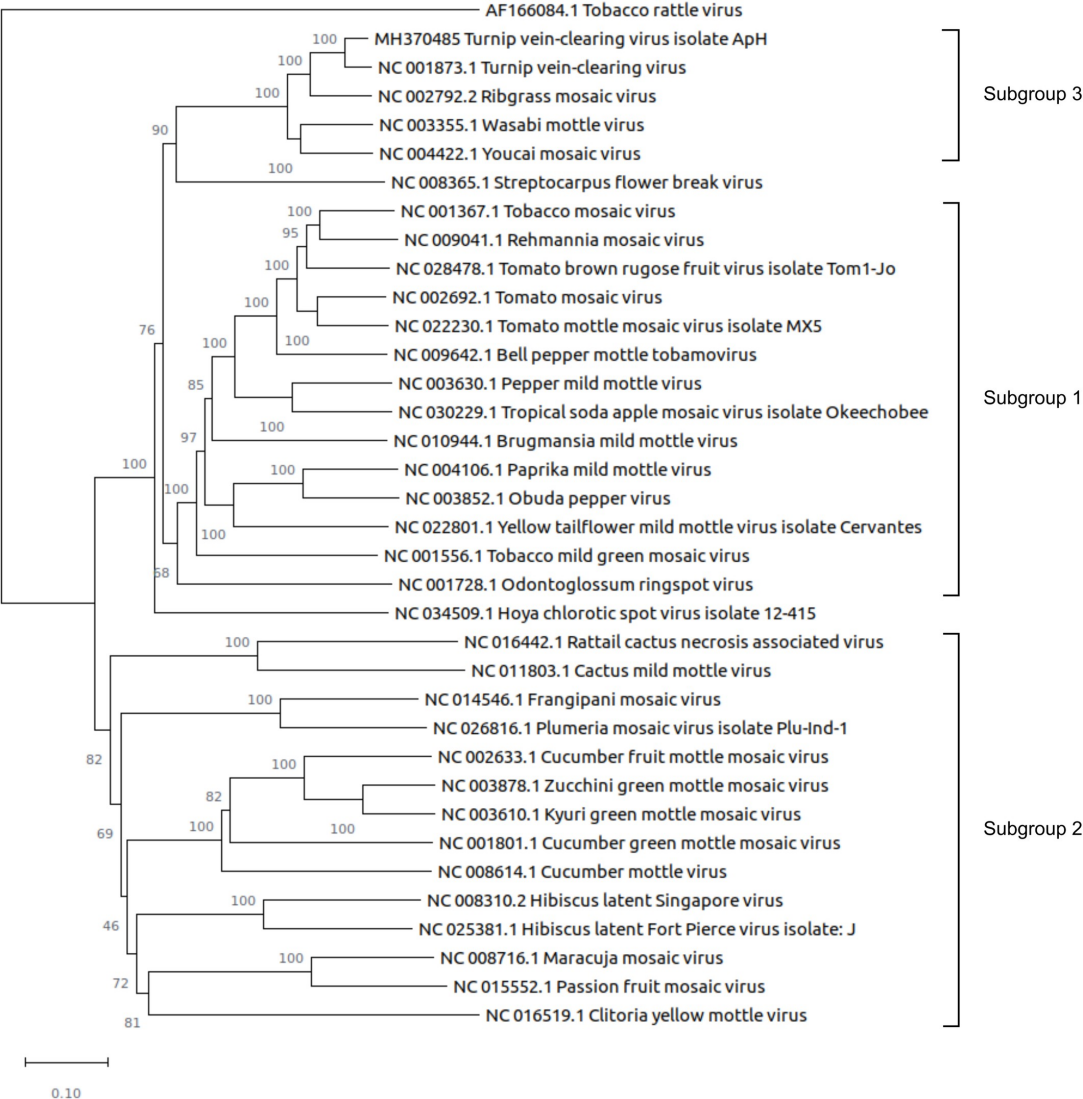
Phylogenetic relationship of the TVCV-ApH and some representative members of tobamoviruses. The evolutionary history was inferred using the Neighbor-Joining method [25]. The optimal tree with the sum of branch length = 8.01862305 is shown. The percentage of replicate trees in which the associated taxa clustered together in the bootstrap test (1000 replicates) is shown next to the branches [27]. All positions containing gaps and missing data were eliminated. There were a total of 5052 positions in the final dataset. Tobacco rattle virus was used as an outgroup.

Phylogenetic analysis of the full-length tobamovirus genomes from the RefSeq database confirmed that the ApH isolate is most closely related to TVCV and belongs to Subgroup 3 of the genus Tobamovirus (Fig 2). Based on the molecular and phylogenetic data we conclude that isolate ApH is a strain of TVCV and propose to name it TVCV-ApH.

### Comparison of TVCV isolates with full genomes

**Fig 3:**
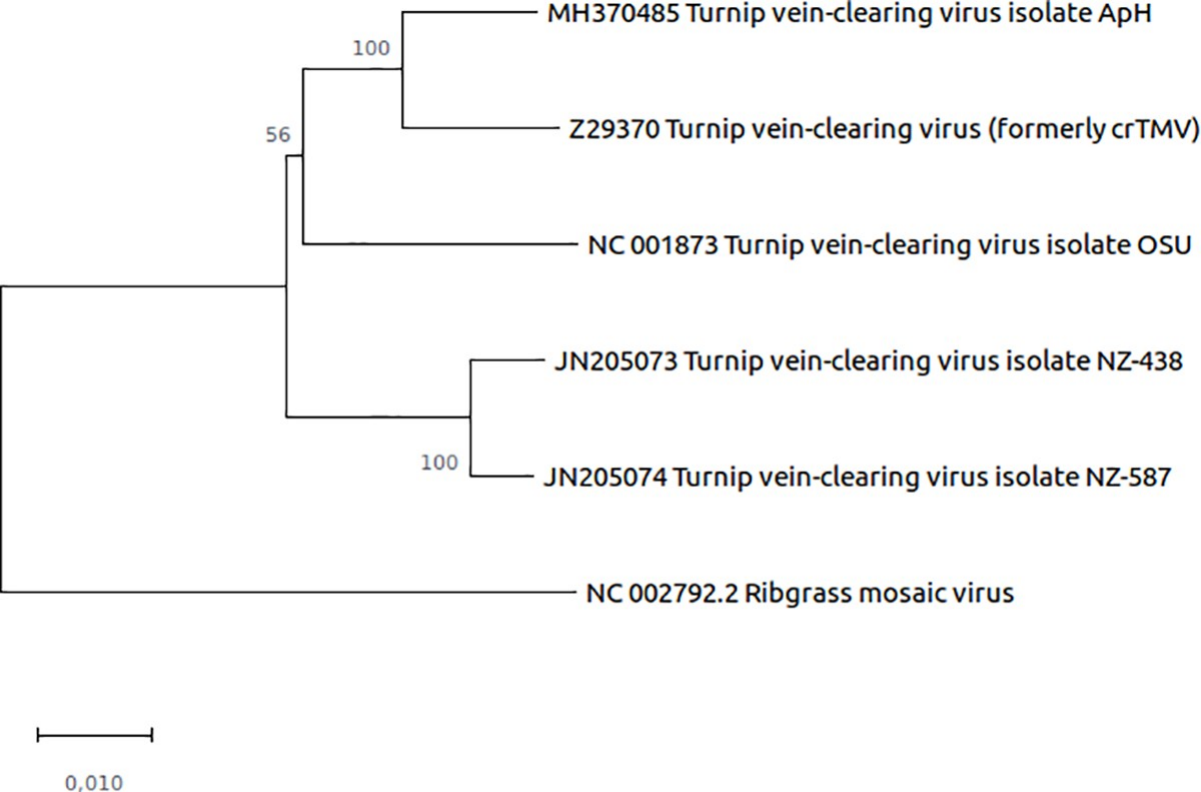
Phylogenetic relationship of the TVCV-ApH and the TVCV isolates with available full genome sequences. The evolutionary history was inferred using the Neighbor-Joining method [25]. The optimal tree with the sum of branch length = 0.16283470 is shown. The percentage of replicate trees in which the associated taxa clustered together in the bootstrap test (1000 replicates) are shown next to the branches [27]. All positions containing gaps and missing data were eliminated. There were a total of 5965 positions in the final dataset. Ribgrass mosaic virus was used as an outgroup.

Currently, four complete TVCV genome sequences are available in the GenBank database (NC_001873.1, Z29370, JN205074.1, JN205073.1). Based on phylogenetic analyses using full genome nucleotide sequences, the closest relative of TVCV-ApH is the isolate that was formerly known as crTMV [12] (Fig 3).

Comparison of the viral-encoded proteins of TVCV-ApH and the four TVCV isolates revealed a moderate amount of differences in the amino acid sequences (S1 Fig).

### Genome organization of TVCV-ApH

**Fig 4:**
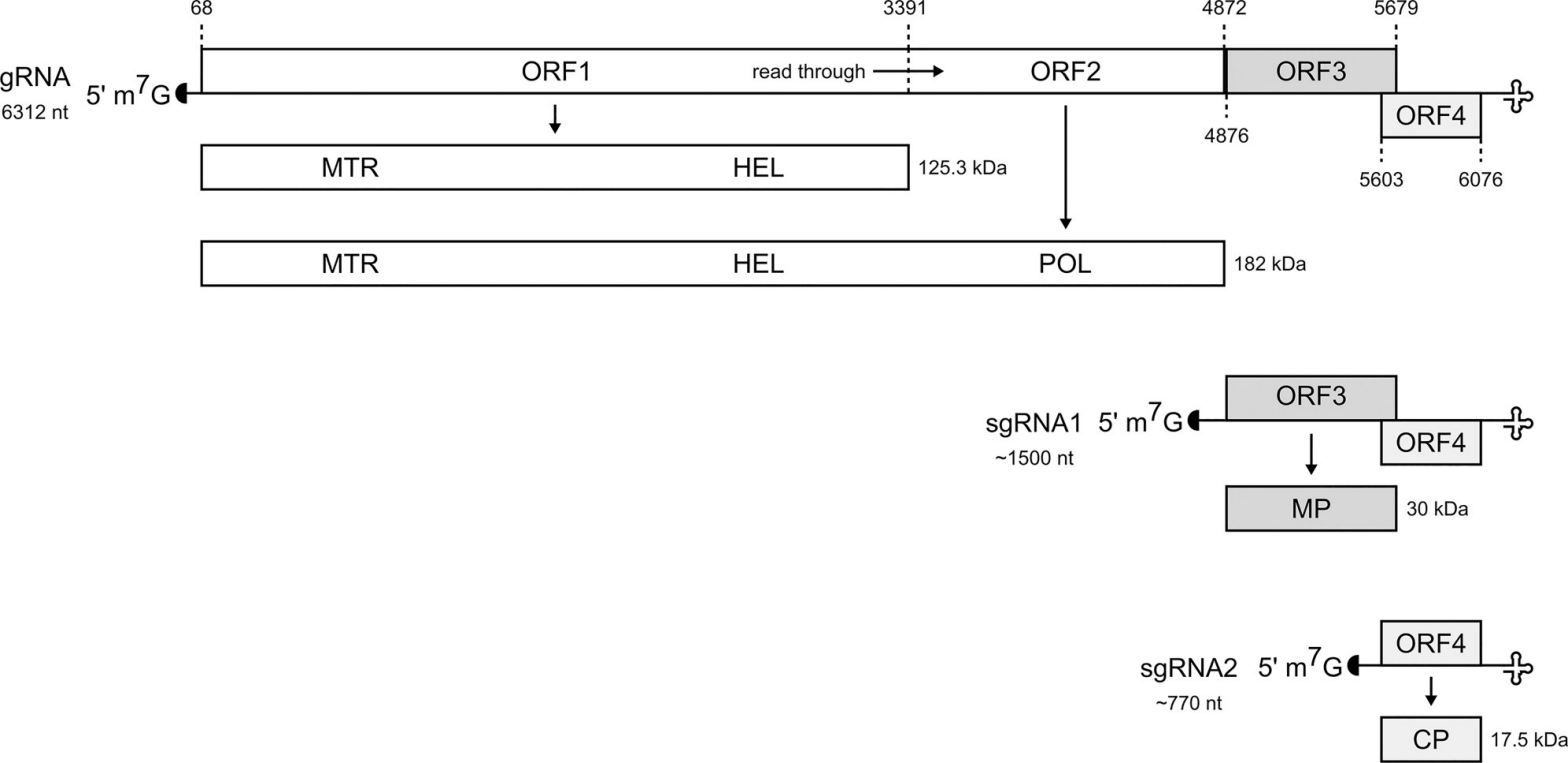
Schematic representation of the genome organization of TVCV-ApH. Similarly to other tobamoviruses, the TVCV-ApH genome contains four ORFs. The genomic RNA is a template for expression of the 125 and 182 kDa replication proteins. The 3’ distal movement protein (MP) and capsid protein (CP) ORFs are expressed from separate 3’ co-terminal sgRNAs. The tRNA structure motif at the 3’-end of the RNA is represented by a cloverleaf motif.

The genome of TVCV-ApH is 6312-nt-long containing four open reading frames (ORF) (Fig 4). The first ORF starts from an AUG at nts 68–70 and terminates with an amber stop codon UAG at nts 3389–3391. Readthrough of the leaky amber termination codon would extend the frame up to a stop codon at nt 4871–4873 (ORF2). ORF1 is 3324-nt-long and encodes a polypeptide of about 125.3 kDa. The ORF1 encoded putative replication protein contains an Alphavirus-like methyltransferase (MTR) domain (position 72–280 aa; PS51743) and (+) RNA virus helicase core domain (position 795–1107 aa; PS51657) [29]. ORF2 is 4806-nt-long (68–4873 nt) and encodes a polypeptide of about 182 kDa. The ORF2 encoded putative replication protein contains an RNA-directed RNA polymerase, catalytic domain (position 1368–1481 aa; PS50507) [29]. ORF3 starts from nt 4876 and terminates with a UAA at nts 5677–5679. ORF3 encodes the putative cell-to-cell movement protein (MP) with a size of ca. 30.1 kDa. ORF4 overlaps with ORF3 and starts from nt 5603 and terminates at nt 6076 and encodes the coat protein with a size of 17.5 kDa. The 5’ untranslated region is 67-nt-long and the 3’ untranslated region is 236-nt-long.

### Characterization of the infectious *in vitro* transcribed viral RNA

**Fig 5:**
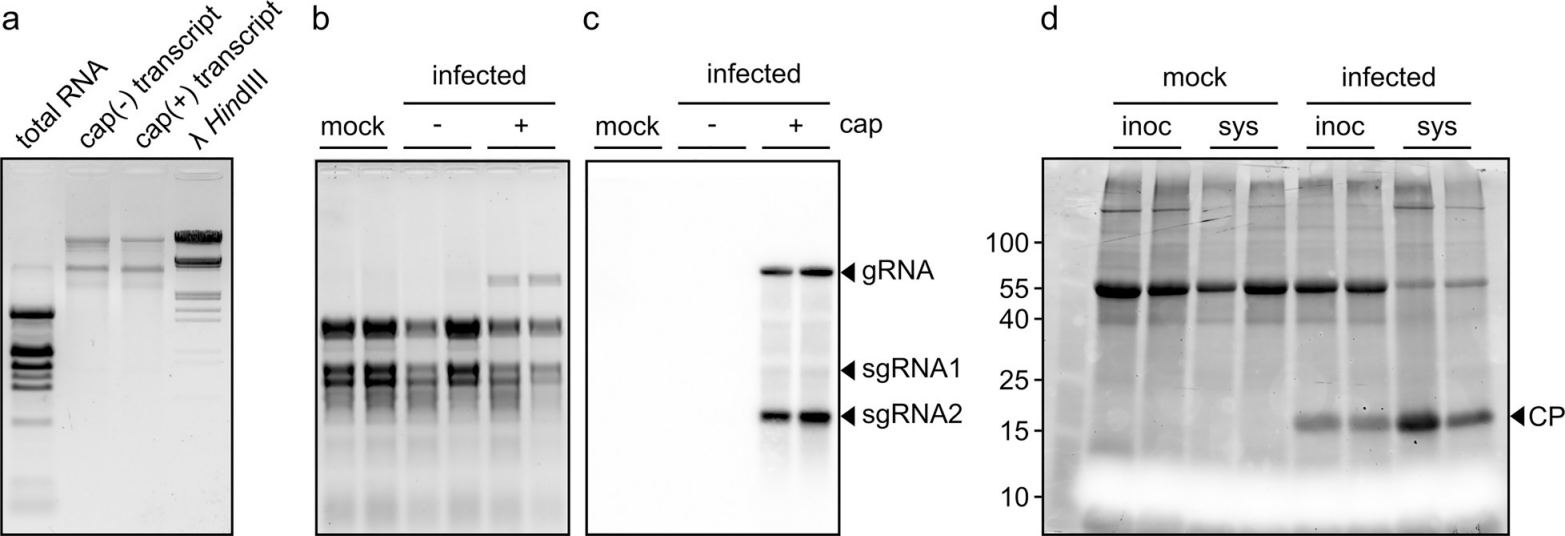
Infection of *Nicotiana benthamiana* plants with the *in vitro* transcribed TVCV-ApH. (a) Agarose gel electrophoresis of *in vitro* synthesized infectious transcripts with or without the m7G cap analog. (b) Denaturing agarose gel electrophoresis of total RNAs from mock-treated, uncapped-, and capped-transcript-treated *Nicotiana benthamiana* plants after 5 dpi. The gel was stained with ethidium bromide. (c) The same gel was blotted to a membrane and subjected to northern blot analysis using a virus-specific probe. The genomic (gRNA) and the subgenomic RNAs (sgRNA1 and sgRNA2) are marked. (d) Total proteins were purified from the inoculated and the systemic leaves of the same mock- and capped-transcript-treated plants as in the case of northern analysis and run on 12% TGX Stain-Free^™^ FastCast^™^ Acrylamide Gels (Bio-Rad). The 18 kDa Coat Protein is marked.

Full-length clone of TVCV-ApH was constructed with a T7 promoter in front of the genomic sequence of the virus (pUC19-T7::TVCV-BstXI) as described in the materials and methods section. The full-length capped and uncapped *in vitro* transcripts of TVCV-ApH were prepared by transcription of *Bst*XI-linearized pUC19-T7::TVCV-BstXI plasmid with T7 RNA polymerase. The *in vitro* transcribed viral RNAs co-migrated with the TVCV-ApH genomic RNA extracted from virus-infected plants (Fig 5A). *In vitro* transcripts of both capped and uncapped TVCV-ApH RNAs were infectious; however, infectivity was greatly reduced and virus accumulation delayed in the absence of the cap structure (Fig 5B and 5C). All plants inoculated either with the wild-type viral RNA or the *in vitro* synthesized, capped TVCV-ApH RNA showed similar symptoms, which developed with the same intensity and rapidity. Furthermore, northern blot analyses confirmed that the patterns of viral-specific genomic and subgenomic RNA species were equivalent to the wild-type virus (Fig 5C). Moreover, SDS-PAGE analyses of the protein extract from *in vitro* transcribed TVCV-ApH RNA-infected plants confirmed the accumulation of a protein of ca. 18 kDa, which is characteristic of TVCV coat protein (Fig 5D). Therefore we concluded that the *in vitro* transcribed TVCV-ApH RNA is biologically active and pathologically indistinguishable from the parent virus.

### Symptom recovery can be observed in plants infected with crTMV but not with TVCV-ApH

ORMV (also called Youcai mosaic virus or Chinese rape mosaic virus, or TMV-Cg) like TVCV, belongs to the Subgroup 3 of the tobamoviruses that causes symptoms in crucifers and is a commonly used virus model for studies in *Arabidopsis thaliana* [30]. It was reported recently, that *A*. *thaliana* plants infected with ORMV undergo natural symptom recovery which is characterized by the emergence of asymptomatic leaves following a systemic symptomatic infection [31]. To test whether TVCV-ApH infection also results in symptom recovery, we inoculated *A*. *thaliana* ecotype Bur-0 plants either with *in vitro* transcripts of TVCV-ApH or crTMV [12], the closest relative of TVCV-ApH, according to our phylogenetic analysis (Fig 3). TVCV-ApH has a 96.15% nucleotide identity (242 SNPs) compared to the crTMV at the full genome level. Multiple alignment of the viral-encoded proteins of the TVCV isolates with full genomes including TVCV-ApH and crTMV is in S1 Fig.

**Fig 6:**
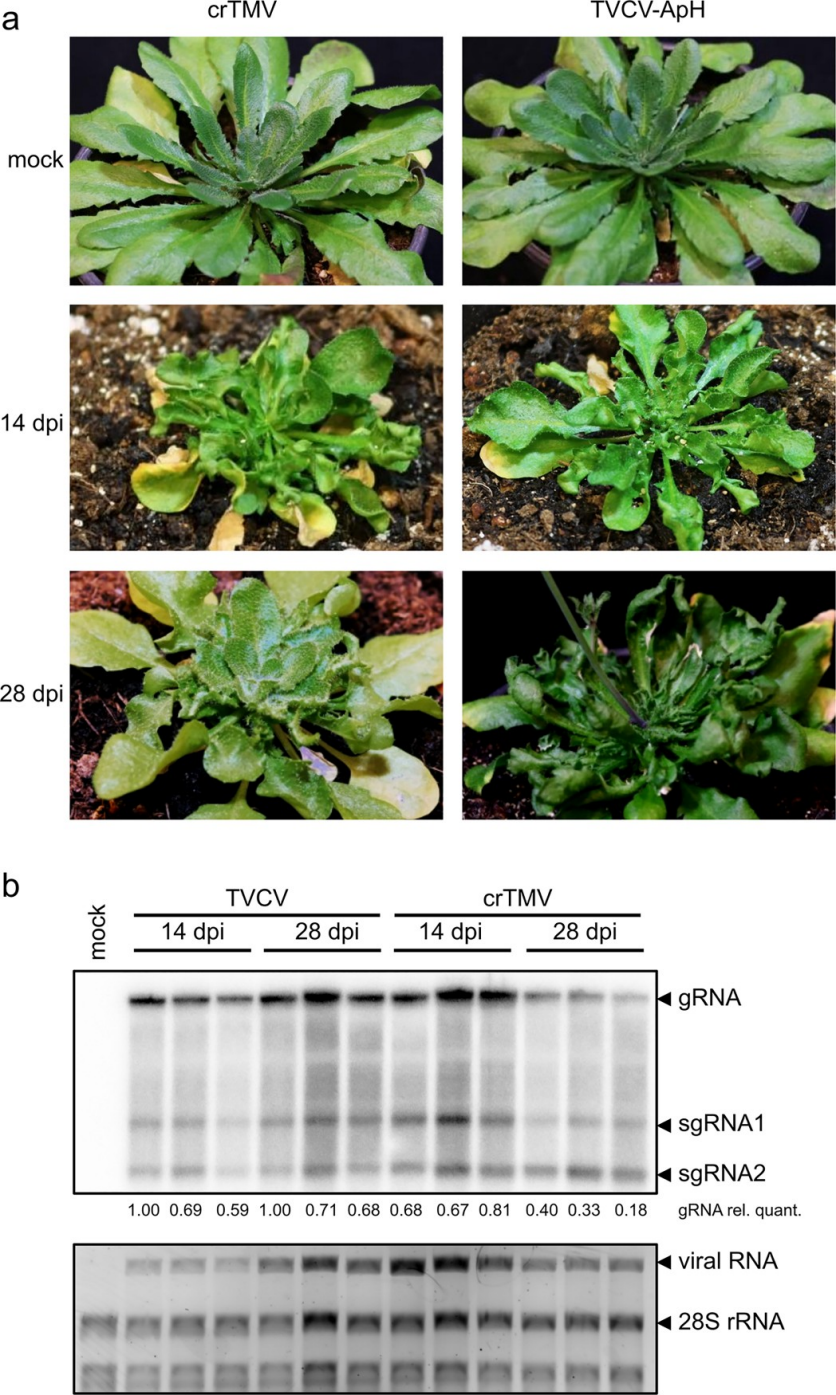
Infection of *Arabidopsis thaliana* Bur-0 plants with TVCV-ApH- and crTMV. (a) Symptom development and recovery in the Bur-0 ecotype of *Arabidopsis thaliana* plants infected either with TVCV-ApH or crTMV. (b) The viral genomic (gRNA) and subgenomic RNA (sgRNA1 and sgRNA2) levels were detected in a northern blot assay using a probe that recognizes the 3’ end of the viruses which is highly conserved at the nucleotide level. The loading was checked by running the total RNAs on an agarose gel that was stained with ethidium bromide. For quantitative analysis, the density of the gRNA northern signal was measured in every sample and was normalized to the 28S ribosomal RNA (28S rRNA) levels in the corresponding sample. The values were further normalized to the first sample. The resulting relative values were placed under the samples.

Both TVCV-ApH- and crTMV-infected plants showed disease symptoms at 14 days post-inoculation (dpi) and equally high level of virus accumulation in the systemically infected leaves by northern blot analysis (Fig 6). Similarly to the ORMV-infected plants reported previously [31], from the fourth week after inoculation, all newly emerging leaves recovered in 9 crTMV-infected plants out of the 10 plants tested, while none of the TVCV-ApH-infected plants recovered. The virus level also remained high in the symptomatic leaves, while decreased in the symptomless ones (Fig 6).

### The infectious clone of TVCV-ApH may serve the scientific community

The available infectious cDNA clone of TVCV-ApH can be useful for further studies to identify viral factors responsible for the marked differences in symptom recovery phenotype between TVCV-ApH and the closely related crTMV.

## Supporting information

S1 Fig Multiple alignment of the virus-encoded proteins in TVCV isolates. Five complete TVCV genomes are available in GenBank including TVCV-ApH (GenBank accession numbers: MH370485, NC_001873.1, Z29370, JN205074.1, JN205073.1). Their annotated proteins were aligned with Clustal Omega and visualized in Jalview. The residues are colored by sequence identity. The red asterisks mark the sites that are different in TVCV-ApH and crTMV. ORF1 is not shown because it is part of ORF2 (see Fig 4).
(PDF)

S1 Raw Images Original, uncropped images of gels and blots presented in figures. (PDF)
