# Supplementary material for: Molecular characterization and In Vitro synthesis of infectious RNA of a Turnip vein-clearing virus isolated from Alliaria petiolata in Hungary
Source: PLoS One. 2019 Oct 24;14(10):e0224398. doi: 10.1371/journal.pone.0224398 (PMC6812821; doi:10.1371/journal.pone.0224398)
Supplement: S1 Fig — Five complete TVCV genomes are available in GenBank including TVCV-ApH (GenBank accession numbers: MH370485, NC_001873.1, Z29370, JN205074.1, JN205073.1). Their annotated proteins were aligned with Clustal Omega and visualized in Jalview. The residues are colored by sequence identity. The red asterisks mark the sites that are different in TVCV-ApH and crTMV. ORF1 is not shown because it is part of ORF2 (see Fig 4). (PDF) [file pone.0224398.s001.pdf]

ORF2 (RNA-dependent RNA Polymerase)

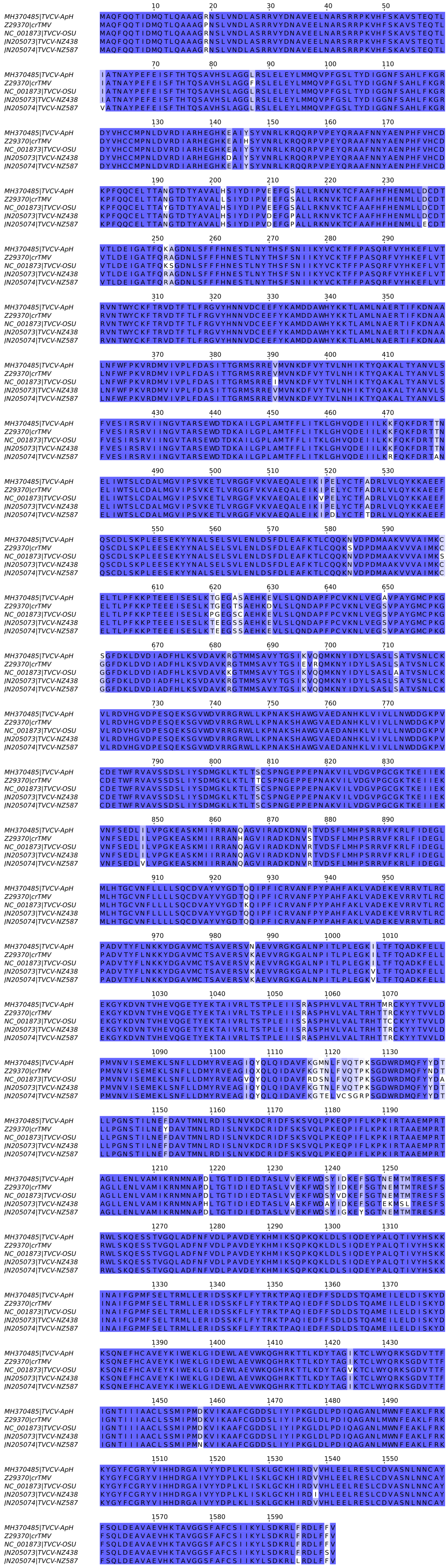

ORF3 (Movement Protein)

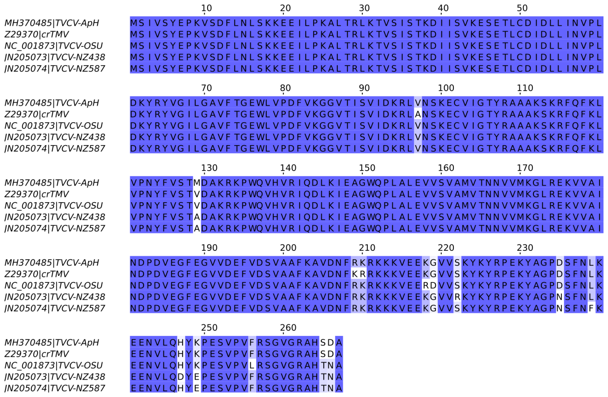

ORF4 (Coat Protein)

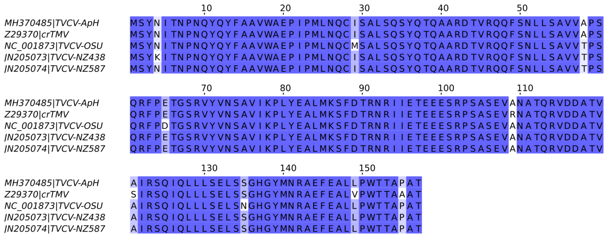

S1 Fig. Multiple alignment of the virus-encoded proteins in TVCV isolates. Five complete TVCV genomes are available in GenBank including TVCV-ApH (GenBank accession numbers: MH370485, NC_001873.1, Z29370, JN205074.1, JN205073.1). Their annotated proteins were aligned with Clustal Omega and visualized in Jalview. The residues are colored by sequence identity. The red asterisks mark the sites that are different in TVCV-ApH and crTMV. ORF1 is not shown because it is part of ORF2 (see Fig 4).
