# Supplementary material for: Molecular characterization and In Vitro synthesis of infectious RNA of a Turnip vein-clearing virus isolated from Alliaria petiolata in Hungary
Source: PLoS One. 2019 Oct 24;14(10):e0224398. doi: 10.1371/journal.pone.0224398 (PMC6812821; doi:10.1371/journal.pone.0224398)
Supplement: S1 Raw Images — (PDF) [file pone.0224398.s002.pdf]

Fig 5a, original, uncropped image

Ethidium bromide stained agarose gel

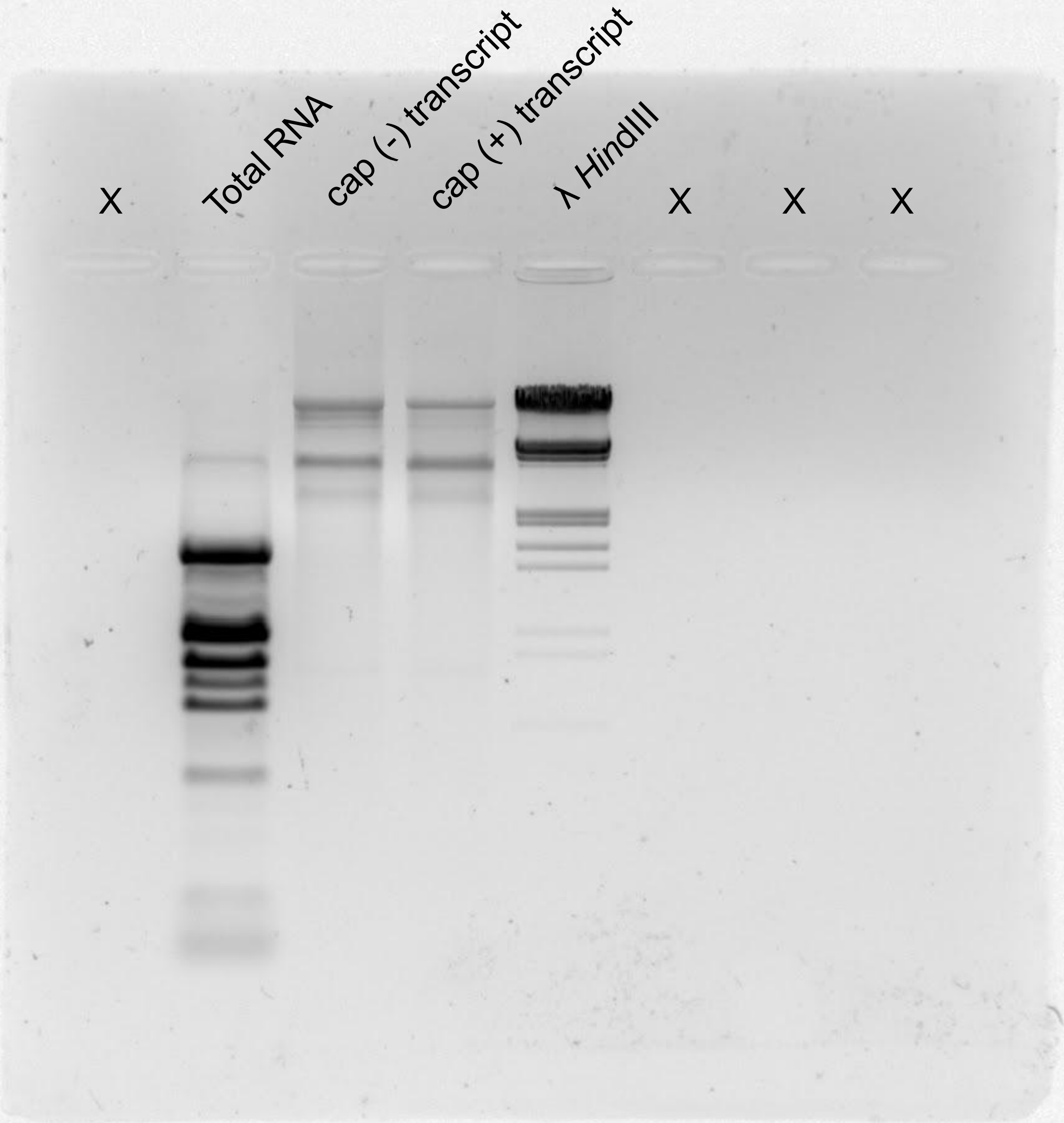

Fig 5b, original, uncropped image

Ethidium bromide stained agarose gel

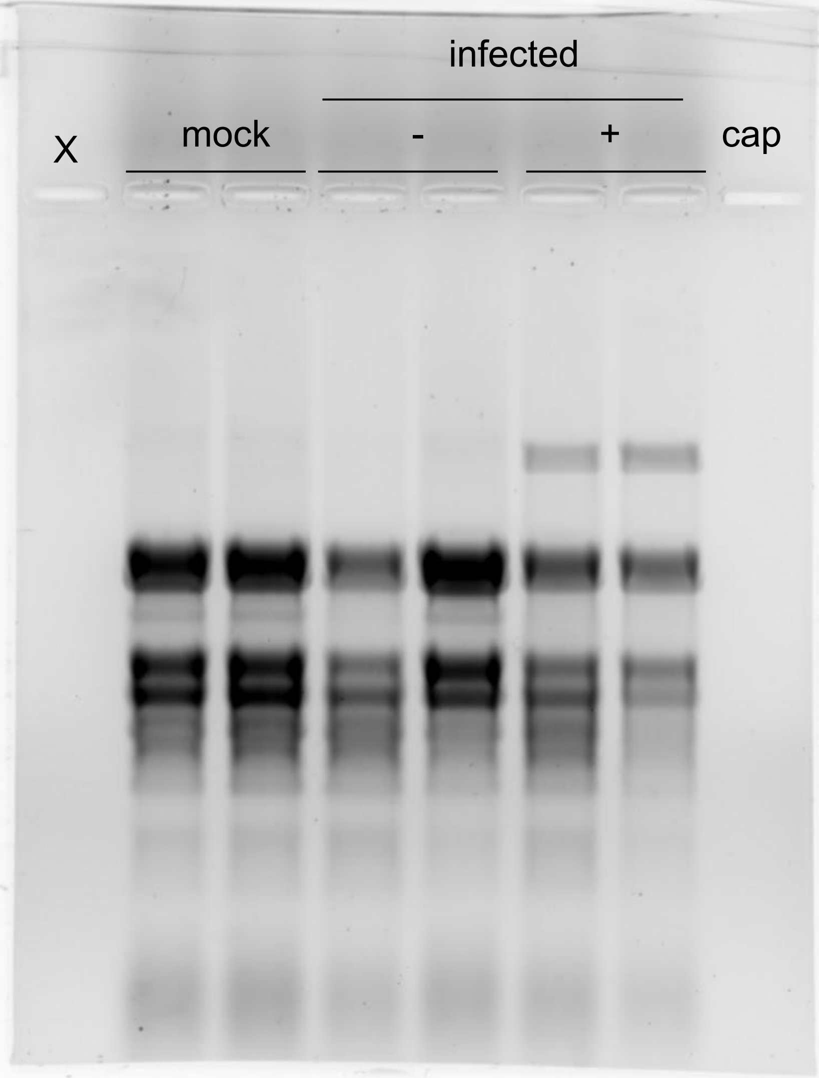

Fig 5c, original, uncropped image

northern blot autoradiogram captured by PhosphorImager

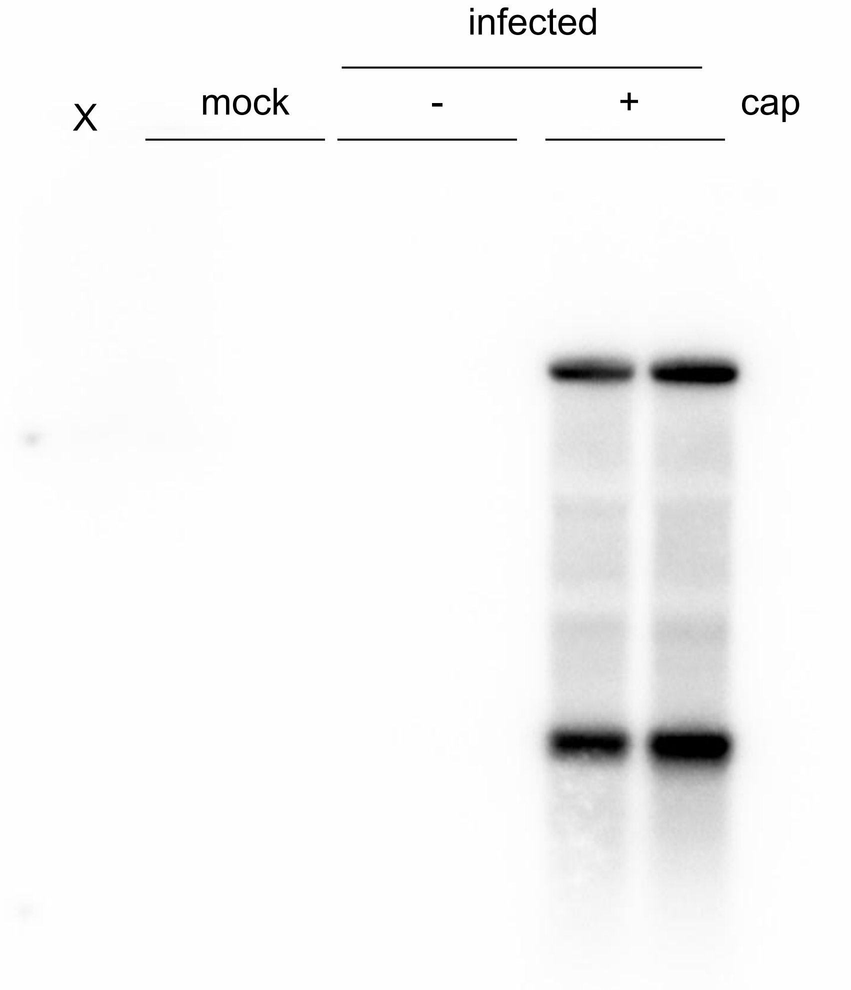

Fig 5d, original, uncropped image

12% TGX Stain-Free™ FastCast™ Acrylamide Gel (Bio-Rad) using ProSieve QuadColor™ protein marker (4.6–300 kDa) (Lonza)

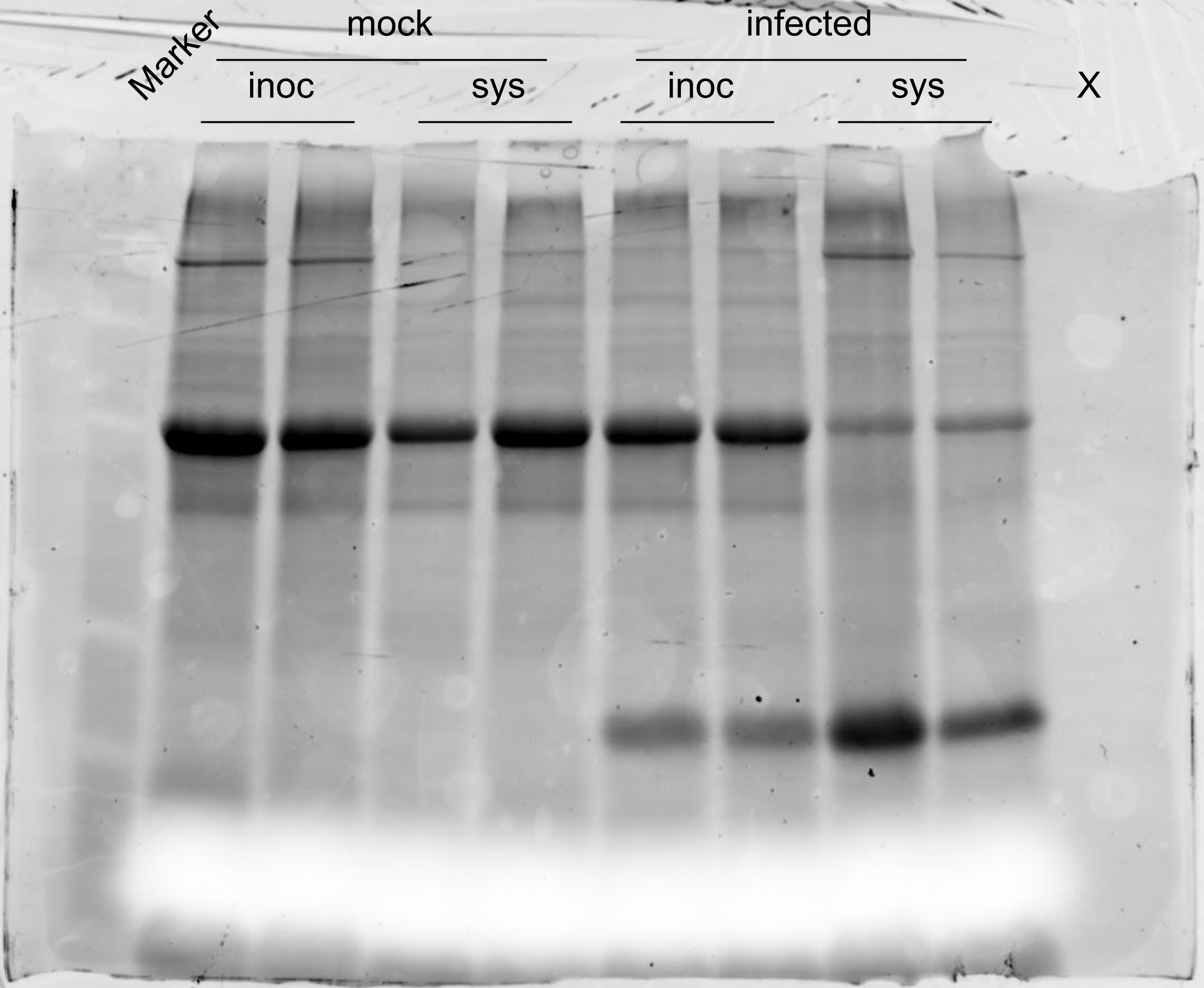

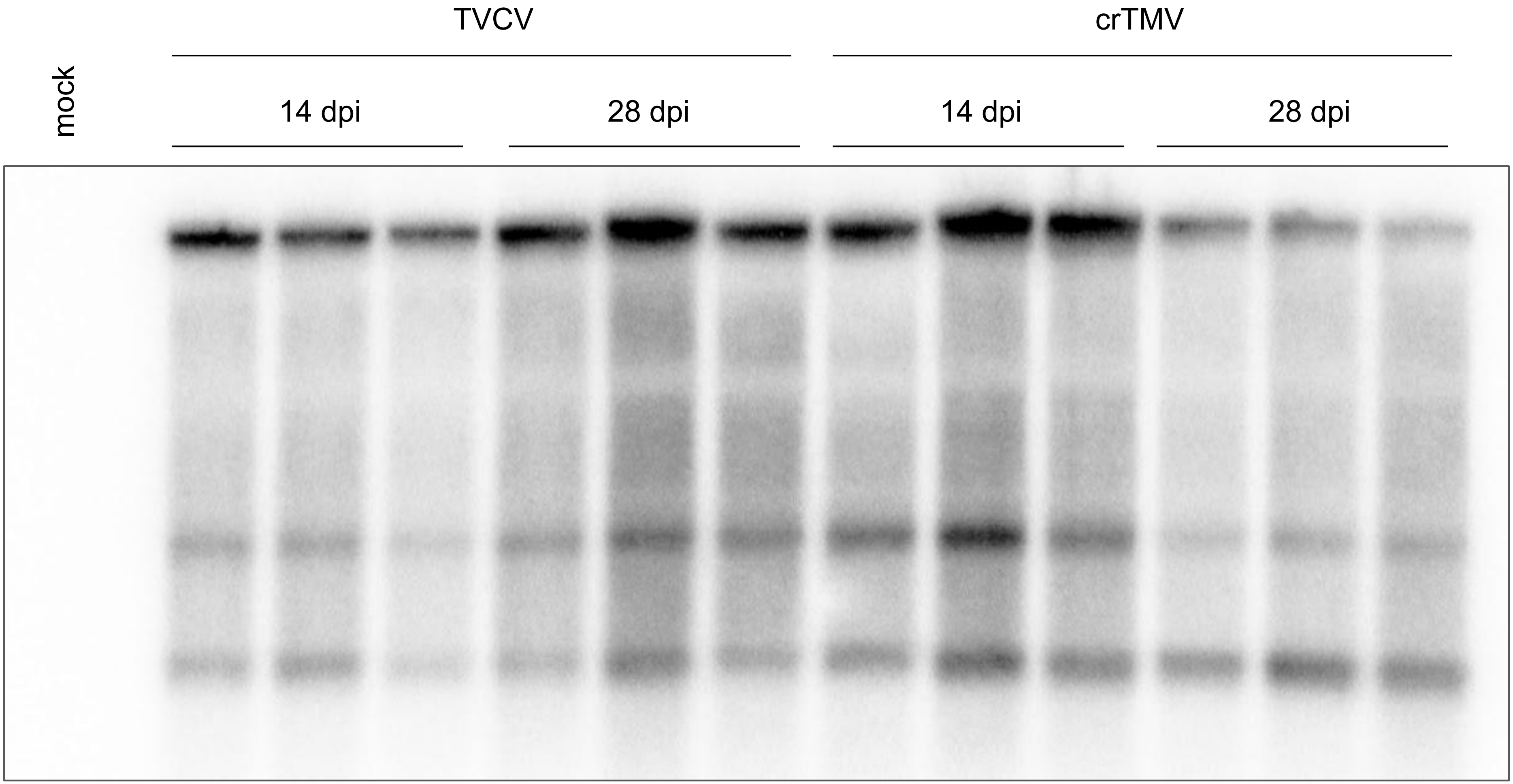

Fig 6b upper row, original, uncropped image

northern blot autoradiogram captured by PhosphorImager

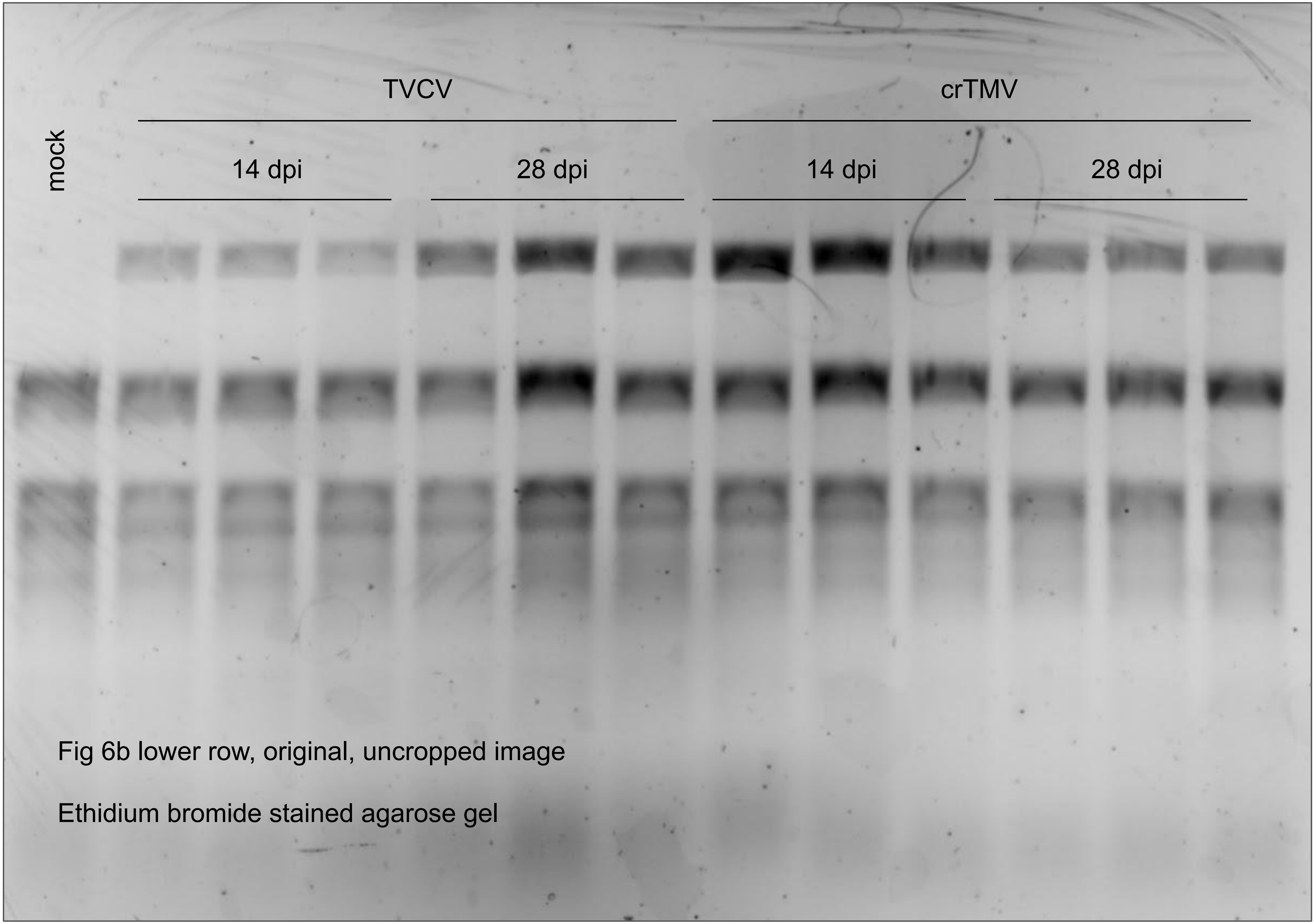

Fig 6b lower row, original, uncropped image

Ethidium bromide stained agarose gel
